# Decursin and Doxorubicin Are in Synergy for the Induction of Apoptosis via STAT3 and/or mTOR Pathways in Human Multiple Myeloma Cells

**DOI:** 10.1155/2013/506324

**Published:** 2013-05-13

**Authors:** Jinsil Jang, Soo-Jin Jeong, Hee-Young Kwon, Ji Hoon Jung, Eun Jung Sohn, Hyo-Jung Lee, Ji-Hyun Kim, Sun-Hee Kim, Jin Hyoung Kim, Sung-Hoon Kim

**Affiliations:** ^1^Cancer Preventive Material Development Research Center, College of Oriental Medicine, Kyung Hee University, 1 Hoegi-dong, Dongdaemun-gu, Seoul 131-701, Republic of Korea; ^2^Basic Herbal Research Group, Korea Institute of Oriental Medicine, Daejeon 305-811, Republic of Korea; ^3^Medical Genomics Research Center, Korea Research Institute of Bioscience and Biotechnology, Daejeon 305-806, Republic of Korea

## Abstract

*Background*. Combination cancer therapy is one of the attractive approaches to overcome drug resistance of cancer cells. In the present study, we investigated the synergistic effect of decursin from *Angelica gigas* and doxorubicin on the induction of apoptosis in three human multiple myeloma cells. *Methodology/Principal Findings*. Combined treatment of decursin and doxorubicin significantly exerted significant cytotoxicity compared to doxorubicin or decursin in U266, RPMI8226, and MM.1S cells. Furthermore, the combination treatment enhanced the activation of caspase-9 and -3, the cleavage of PARP, and the sub G1 population compared to either drug alone in three multiple myeloma cells. In addition, the combined treatment downregulated the phosphorylation of mTOR and its downstream S6K1 and activated the phosphorylation of ERK in three multiple myeloma cells. Furthermore, the combined treatment reduced mitochondrial membrane potential, suppressed the phosphorylation of JAK2, STAT3, and Src, activated SHP-2, and attenuated the expression of cyclind-D1 and survivin in U266 cells. Conversely, tyrosine phosphatase inhibitor pervanadate reversed STAT3 inactivation and also PARP cleavage and caspase-3 activation induced by combined treatment of doxorubicin and decursin in U266 cells. *Conclusions/Significance*. Overall, the combination treatment of decursin and doxorubicin can enhance apoptotic activity via mTOR and/or STAT3 signaling pathway in multiple myeloma cells.

## 1. Introduction

Multiple myeloma is a neoplasm of terminally differentiated neoplastic B cells (plasma cells), accounting for about 10% of all hematologic malignancies [1–3]. Multiple myeloma is characterized by the infiltration and accumulation of monocytic plasma cells in the bone marrow [4]. Although various chemotherapeutic agents such as thalidomide [5], bortezomib [6], and lenalidomide [7] are successful in initial chemotherapy of multiple myeloma, the patients ultimately become drug resistance [4].

Recently, many groups reported that combination therapy is an effective process to overcome drug resistance of various types of human malignant diseases, including multiple myeloma. For instance, Stein and colleagues reported that milatuzumab, a humanized anti-CD74 monoclonal antibody, improved the response of multiple myeloma to treatment with bortezomib, doxorubicin, or dexamethasone [8]. Chauhan and colleagues demonstrated that 2-methoxyestradiol (2-ME), an estrogen derivative, enhanced dexamethasone-induced apoptosis [9]. In addition, Sanchez and colleagues showed that the proteasome inhibitor CEP-18770 was able to augment the antimyeloma activity of bortezomib and melphalan [10].

*Angelica gigas *Nakai (Umbelliferae) has been used in traditional oriental medicine for the prevention and treatment of blood diseases including anemia as a tonic agent [11]. Its major compound decursin exerted antitumor activity by apoptosis induction or angiogenesis inhibition in various cancer cells including prostate, bladder, and colon cancer, and leukemia [12–14]. Also, our group reported that decursin has a protective effect on neurotoxicity and nephrotoxicity in normal cells via activation of antioxidative enzymes [15, 16] and also decursin-induced apoptosis through inhibition of STAT3 signaling pathway in multiple myeloma U266 cells [17]. 

Also, the mammalian target of rapamycin (mTOR), a serine/threonine (ser/thr) protein kinase, regulates cell growth mediated by interaction of signals from growth factors, and its downstream protein ribosomal S6 kinas (S6 K1) also plays a crucial role in cell cycle progression. Thus, inhibition of mTOR pathway to induce apoptosis is an attractive target for cancer therapy [18–20]. 

**Figure 1:**
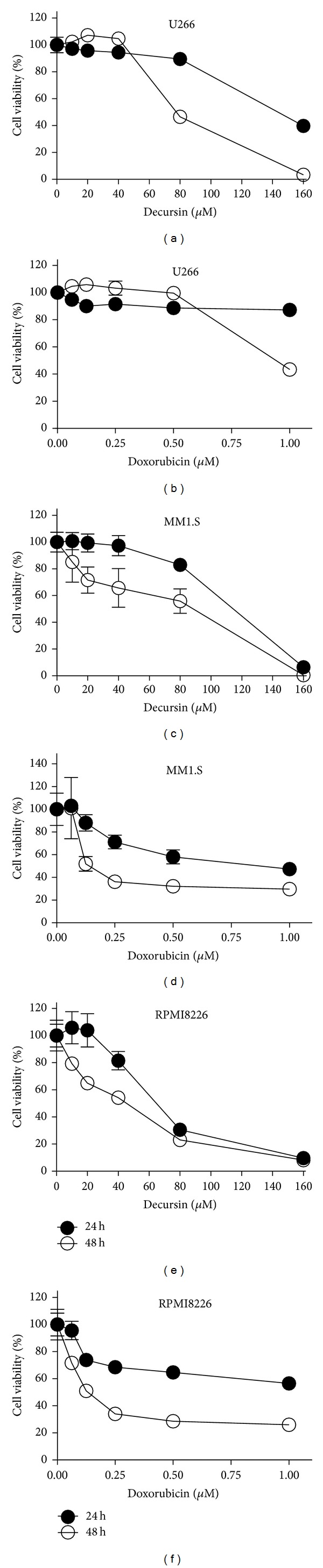
Cytotoxic effects of doxorubicin and decursin against multiple myeloma cells. (a) U266 cells were treated with various concentrations of decursin (0, 10, 20, 40, 80, or 160 *μ*M) for 24 h or 48 h. (b) U266 cells were treated with various concentrations of doxorubicin (0, 0.0625, 0.125, 0.25, 0.5, or 1 *μ*M) for 24 h or 48 h. (c) MM.1S cells were treated with various concentrations of decursin (0, 10, 20, 40, 80 or 160 *μ*M) for 24 or 48 h. (d) MM.1S cells were treated with various concentrations of doxorubicin (0, 0.0625, 0.125, 0.25, 0.5, or 1 *μ*M) for 24 or 48 h. (e) RPMI8226 cells were treated with various concentrations of decursin (0, 10, 20, 40, 80, or 160 *μ*M) for 24 or 48 h. (f) RPMI8226 cells were treated with various concentrations of doxorubicin (0, 0.0625, 0.125, 0.25, 0.5, or 1 *μ*M) for 24 or 48 h. Cytotoxicity of doxorubicin or decursin was evaluated by XTT assay. Data are presented as mean ± SD for triplicate experiments.

Thus, in the present study, we investigated the synergistic effects of decursin and doxorubicin combination (Figure 1) for multiple myeloma treatment. The concurrent treatment of decursin and doxorubicin switched on mitochondria-governed, apoptotic machinery in multiple myeloma cells. In addition, we suggest that combination of decursin- and doxorubicin-induced apoptosis through the suppression of the mTOR/S6K1 and, or STAT3 signaling pathway.

## 2. Materials and Methods

### 2.1. Reagents

Decursin was extracted and purified as described previously [21, 22]. The purity was determined to be ~98.6%. Doxorubicin hydrochloride was purchased from Sigma (St. Louis, MO, USA). Both decursin and doxorubicin were dissolved in dimethyl sulfoxide (DMSO). In all experiment, DMSO concentration was kept below 0.2% (v/v) to remove the effect of vehicle DMSO.

### 2.2. Cell Culture

U266 [23], MM.1S [24], and RPMI8226 cells were obtained from American Type Culture Collection and maintained in RPMI 1640 supplemented with 10% fetal bovine serum (FBS, Welgene, Korea), nonessential amino acids, pyruvate, glutamine, vitamins, penicillin (100 U/mL), and streptomycin (100 g/mL). The cells were routinely tested for mycoplasma contamination to ensure that only contaminated negative cells were used.

### 2.3. Cytotoxicity Assay

The cytotoxicity of decursin and/or doxorubicin was measured by 2,3-bis-(2-methoxy-4-nitro-5-sulfophenyl)-2*H*-tetrazolium-5-carboxanilide (XTT) colorimetric assay (Sigma Chemical Co., St. Louis, Mo, USA). U266, RPMI8226 or MM1.S cells were seeded onto 96-well microplates at a density of 2 × 10^4^ cells per well in 100 *μ*L of growth medium with various concentrations of decursin and/or doxorubicin. XTT working solution was prepared just prior to culture application by mixing 1 mL of XTT stock solution (1 mg/mL in PBS) with 10 *μ*L of phenazine methosulfate (PMS) (1.53 mg/mL in PBS). After incubation at 37°C in a humidified incubator for 24 h, a 50 *μ*L of XTT working solution was added to each well. Cells were incubated at 37°C for 2 h, and the optical density was measured using microplate reader (Sunrise, TECAN) at 450 nm. Cell viability was calculated as a percentage of viable cells in drug-treated group versus untreated control by a following equation. Cell viability (%) = [OD (drug) − OD (Blank)]/[OD (Control)  − OD (Blank)] × 100. Wells containing XTT reagent in the absence of cells were included to verify that the reagent did not interfere with the test.

### 2.4. Combination Index (CI) Calculation

Cells were treated with decursin (40 *μ*M or 80 *μ*M) and doxorubicin (0.5 or 1 *μ*M). The CI was determined by the Chou-Talalay method and CalcuSyn software (Biosoft, Ferguson, MO, USA). A CI of less than 1 was considered synergistic based on Zhao's principle [25]. 

### 2.5. Live/Dead Assay

To measure apoptosis, we used the live and dead assay (Molecular Probes), which determines intracellular esterase activity and plasma membrane integrity. In brief, 1 × 10^6^ cells were incubated with decursin and/or doxorubicin for 24 h. Cells were stained with the live and dead reagent (5 *μ*M ethidium homodimer and 5 *μ*M calcein-AM) and then incubated at 37°C for 30 min. Cells were analyzed under Axio vision 4.0 fluorescence microscope (Carl Zeiss Inc., USA).

### 2.6. Cell Cycle Analysis

To determine apoptosis, cell cycle analysis was performed as previously described [26]. Cells (1 × 10^6^) treated with decursin and/or doxorubicin were harvested, washed twice with cold PBS, and fixed in 75% ethanol at −20°C. The fixed cells were resuspended in 100 *μ*L of PBS containing 10 *μ*L of RNase A (10 mg/mL) and incubated for 1 h at 37°C. The cells were stained by adding 400 *μ*L of propidium iodide (50 *μ*g/mL) for 30 min at room temperature in the dark. The DNA contents of stained cells were analyzed using CellQuest Software with the FACSCalibur (Becton Dickinson, Heidelberg, Germany) flow cytometry.

### 2.7. TdT-Mediated dUTP Nick End Labelling (TUNEL) Assay

Flow cytometric analysis was carried out using an *in situ* cell death detection reagent (Roche Molecular Biochemicals, Mannheim, Germany) as described by the manufacturer. U266 cells (1 × 10^6^) were treated with decursin and/or doxorubicin for 24 h at 37°C. The cells were fixed in 4% paraformaldehyde in PBS at room temperature for 60 min then washed in PBS and permeability enhanced by treatment with 0.1% Triton X-100 in 0.1% sodium citrate for 2 min on ice. Cells were washed twice in PBS and resuspended in TUNEL reaction mixture with TUNEL enzyme and incubated for 60 min at 37°C in a humidified atmosphere in the dark. Cells were washed three times with PBS and analysed by flow cytometry (FACSCalibur, BD Biosciences).

### 2.8. Western Blotting

Cells (1 × 10^6^) treated with decursin and/or doxorubicin were harvested and washed with cold PBS. Cell pellets were lysed in 30 *μ*L of lysis buffer (50 mM Tris-HCl, pH 7.4, 150 mM NaCl, 1% Triton X-100, 0.1% SDS, 1 mM EDTA, 1 mM Na_3_VO_4_, 1 mM NaF, and protease inhibitor cocktail) for 30 min on ice. Lysates were centrifuged at 13,000 ×g for 20 min at 4°C, and the protein contents in the supernatants were measured by using a Bio-Rad detergent compatible (DC) protein assay kit II. Proteins (20 *μ*g/well) were separated by electrophoresis on 4–12% NuPAGE Bis-Tris gels. The proteins then was transferred to Hybond ECL transfer membrane and analyzed with anti-PARP, caspase-8, caspase-9, and caspase-3 antibodies. Protein contents were normalized by reprobing the same membrane with anti-*β*-actin antibody (Sigma).

### 2.9. Measurement of Mitochondrial Membrane Potential

Mitochondrial potential was determined as previously described [27]. U266 cells treated with decursin and/or doxorubicin were incubated for 24 h at 37°C and harvested. After washing twice with cold PBS, the pellets were resuspended in 1 mL of 150 *μ*M TMRE and incubated for 30 min at 37°C in the dark. The fluorescent intensities of cells were analyzed by flow cytometry (FACSCalibur, BD).

### 2.10. Statistical Analysis

All data were expressed as means ± SD of three independent experiments. The statistically significant differences between untreated control and decursin/doxorubicin treated groups were calculated by Student's *t*-test.

## 3. Results

### 3.1. Decursin and Doxorubicin Synergistically Enhanced the Cytotoxic Effect in Multiple Myeloma Cells

To evaluate the cytotoxic effect of decursin or doxorubicin, XTT assay was performed in human multiple myeloma (U266, MM.1S, and RPMI8226). Decursin did not influence the viability of U266 and MM1.S cells up to 80 *μ*M for 24 h culture (Figures 1(a) and 1(c)), while decursin showed significant cytotoxicity in all cells such as U266, MM1.S, and RPMI8226 cells at 80 *μ*M for 48 h culture (Figures 1(a), 1(c) and 1(e)). Doxorubicin at 1 *μ*M had a minimal cytotoxic effect for 24 h and decreased the viability only for 48 h culture in U266 cells (Figure 1(b)), while MM1.S and RPMI8226 cells were more sensitive to doxorubicin at 250 nM than U266 cells after 48 h culture (Figures 1(d) and 1(f)). 

**Figure 2:**
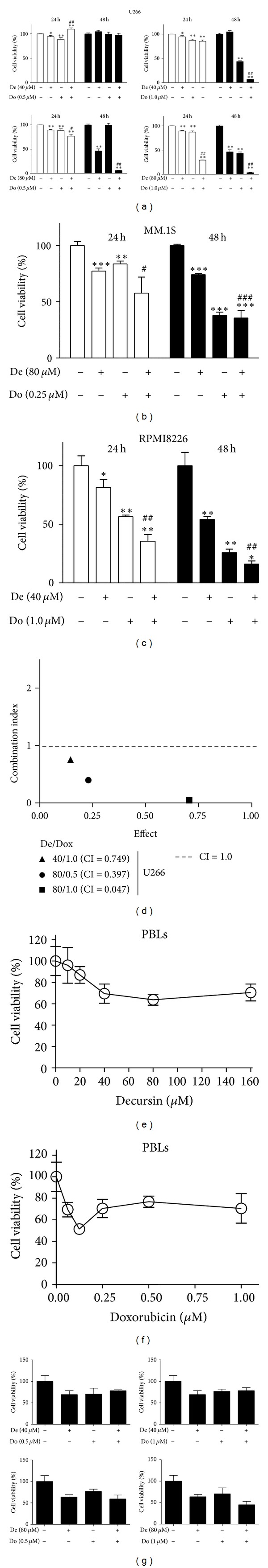
Synergistic effect of decursin and doxorubicin on the cytotoxicity in multiple myeloma cells. (a) U266 cells were treated with decursin (40 or 80 *μ*M) and/or doxorubicin (0.5 or 1 *μ*M) for 24 or 48 h. (b) MM.1S cells were treated with decursin (80 *μ*M) and/or doxorubicin (0.25 *μ*M) for 24 or 48 h. Cytotoxicity of doxorubicin and/or decursin was evaluated by XTT assay. (c) RPMI8226 cells were treated with decursin (40 *μ*M) and/or doxorubicin (1 *μ*M) for 24 or 48 h. Data are presented as means ± SD for triplicate experiments. *, *P* < 0.05, **; *P* < 0.01 and ***; *P* < 0.001 versus untreated control. ^#^, *P* < 0.05, ^##^; *P* < 0.01, ^###^, *P* < 0.001 versus doxorubicin-treated cells. (d) The combination index (CI) between decursin and doxorubicin (24 h) was determined by the Chou-Talalay method and CalcuSyn software (Biosoft, Feruson, MO, USA) in U266 and MM.1S cells. Normal peripheral blood leukocytes (PBLs) were treated with various concentrations of (e) decursin (10, 20, 40, 80, 160 *μ*M) and/or (f) doxorubicin (0, 0.0625, 0.125, 0.25, 0.5 or 1 *μ*M) for 24. (g) Cytotoxicity of decursin (40 *μ*M or 80 *μ*M) and doxorubicin (0.5 or 1 *μ*M) in PBLs was evaluated by XTT assay. Data are presented as mean ± SD for triplicate experiments.

To examine the synergistic activity of decursin and doxorubicin, U266 cells were treated with decursin (40 or 80 *μ*M), doxorubicin (0.5 or 1 *μ*M), or both for 24 or 48 h. As shown in Figure 2(a), the cotreatment of decursin (80 *μ*M) and doxorubicin (1 *μ*M) for 24 h significantly decreased the viability (71%) of U266 cells compared with that treated with doxorubicin (13%) or decursin (11%) alone (Figure 2(a)). A severe cytotoxic effect (>90% cell death) was observed in combination of decursin and doxorubicin (40 *μ*M/1 *μ*M, 80 *μ*M/0.5 *μ*M and 80 *μ*M/1 *μ*M) (Figure 2(a)). Likewise, cotreatment of decursin and doxorubicin (80 *μ*M/0.25 *μ*M, and 40 *μ*M/1 *μ*M, resp.) enhanced the cytotoxicity in MM1.S and RPMI8226 cells (Figures 2(b) and 2(c)) with statistical significance using combination index (CI value = 0.749, 0.397, and 0.047 at 40 *μ*M/1 *μ*M, 80 *μ*M/0.5 *μ*M, and 80 *μ*M/1 *μ*M, resp.) in U266 cells (Figure 2(d)). However, decursin and/or doxorubicin showed weak cytotoxicity against normal peripheral blood leukocytes (PBLs) (Figures 2(e), 2(f), and 2(g)).

### 3.2. Doxorubicin and Decursin Drastically Induced Apoptosis in Multiple Myeloma Cells

**Figure 3:**
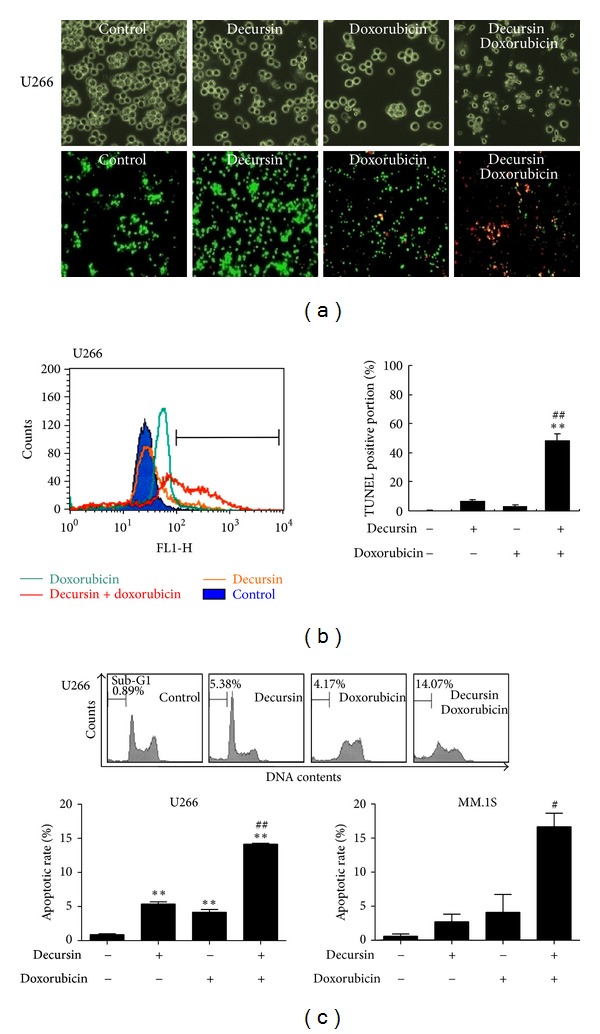
Effect of decursin on doxorubicin-induced apoptosis in multiple myeloma cells. U266 or MM.1S cells were treated with decursin (80 *μ*M) and/or doxorubicin (1 *μ*M) for 24 h. (a) Top panel: morphological changes of U266 cells were observed under inverted microscope (×200). Bottom panel: U266 cell death was determined by the calcein-AM-based live/dead assay as described in Section 2. Red highlights are dead cells, and green highlights live cells. Percent of dead cells was represented. (b) U266 cells were labelled with TdT-mediated dUTP nick end labeling (TUNEL) and analyzed by flow cytometry. (c) U266 or MM.1S cells were stained with PI after fixing in 75% ethanol. DNA contents of sub-G1 were analyzed by flow cytometry. *A representative* result of three independent experiments is shown for each experiment. Data are presented as means ± SD for triplicate experiments. *, *P* < 0.05 and **, *P* < 0.01 versus untreated control. ^#^, *P* < 0.05 and ^##^, *P* < 0.01 versus doxorubicin-treated cells.

We observed after being exposed to decursin, doxorubicin, or both, some morphological changes of U266 cells were observed under a microscopy by live and dead assay (Figure 3(a)). The cotreated cells appeared to swell and with apoptotic shrinkage. To further confirm whether loss of the viability of the cells cotreated with decursin and doxorubicin was due to apoptosis, TUNEL, and live/dead assays were performed in U266 cells. The addition of decursin or doxorubicin alone had a minimal apoptotic effect on the cells. A similar result was obtained from TUNEL assay, in which the numbers of TUNEL-positive cells were significantly increased after the combination treatment (Figure 3(b)), while a few TUNEL-positive cells were detected after the addition of decursin or doxorubicin alone. Consistent with the above results, the co-treatment increased the population of sub-G1 DNA contents (14.07%) compared to decursin (5.38%) or doxorubicin (4.17%) alone (Figure 3(c)), suggesting that low doses of decursin and doxorubicin act in synergy for the induction of apoptosis in U266 cells. Similarly, in MM.1S cells, the cotreatment of decursin and doxorubicin remarkably induced apoptosis (sub-G1; 16.67%) compared to decursin (3.2%) or doxorubicin (4.8%) alone.

### 3.3. Doxorubicin and Decursin Induced Mitochondria-Dependent Apoptosis in Multiple Myeloma Cells

**Figure 4:**
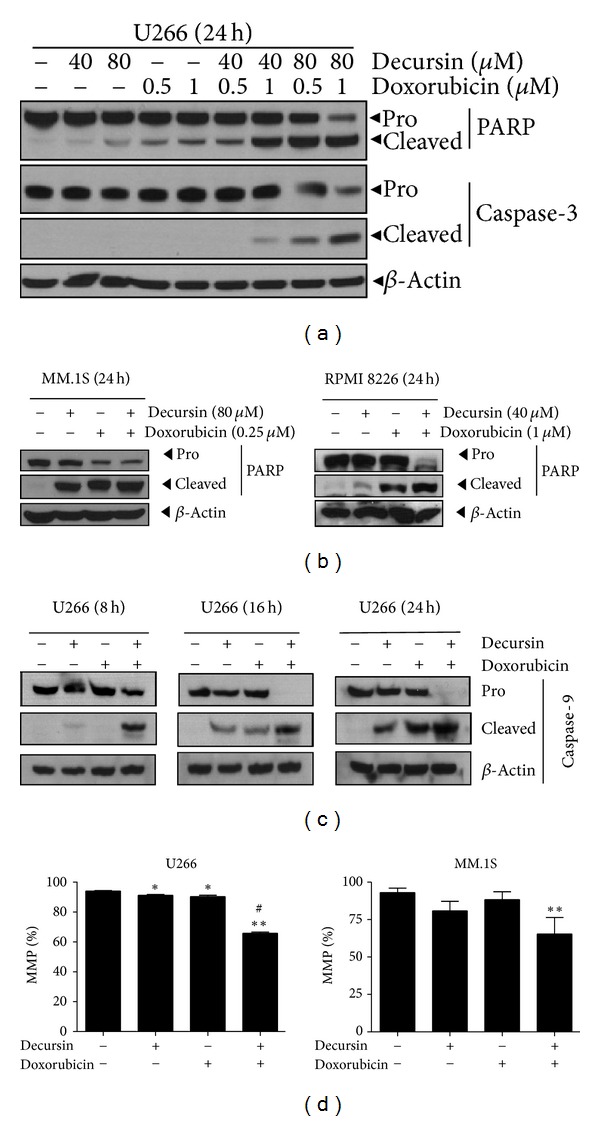
Effect of decursin on mitochondria-dependent apoptosis in doxorubicin-treated multiple myeloma cells. (a) U266 cells were treated with decursin (40 or 80 *μ*M) and/or doxorubicin (0.5 or 1 *μ*M) for 24 h and subjected to western blotting for PARP and caspase-3. (b) MM.1S and RPMI8226 cells were treated with decursin (40, 80 *μ*M, resp.) and/or doxorubicin (0.25, 1 *μ*M, resp.) for 24 h. Western blotting was performed for PARP. (c) U266 cells were treated with decursin (80 *μ*M) and/or doxorubicin (1 *μ*M) for 8 or 16 h, and with decursin (40 or 80 *μ*M) and/or doxorubicin (0.5 or 1 *μ*M) for 24 h. Western blotting was performed for caspase-9. (d) U266 or MM.1S cells were treated with decursin (80 *μ*M) and/or doxorubicin (0.5 or 1 *μ*M) for 24 h and stained with tetramethylrhodamine ethyl ester (TMRE) for 30 min at 37°C, and mitochondrial membrane potential (MMP) was detected by flow cytometry. Graphs represent the percentages of MMP. Data are presented as means ± SD for triplicate experiments. *, *P* < 0.05 and **, *P* < 0.01 versus untreated control. ^#^, *P* < 0.05, versus doxorubicin-treated cells.

Mitochondria plays a crucial role in the regulation of the induction of caspase-dependent and -independent apoptosis [28]. Thus, we examined whether apoptosis induced by decursin plus doxorubicin is mediated through caspase activation. The cotreatment induced a high level of cleaved caspase-3, an effector caspase, PARP (a substrate of caspase-3), compared with that treated with decursin or doxorubicin alone in U266 cells, MM1.S and RPMI8226 cells (Figures 4(a) and 4(b)). Furthermore, cleavage of caspase-9 was observed in three multiple myeloma cells by the combination of decursin and doxorubicin in a time-dependent manner in U266 cells (Figure 4(c)). Consistently, the apoptosis induction was blocked in pretreatment with caspase-9 inhibitor, but not caspase-8 inhibitor (data not shown). These results suggest that the combination of decursin and doxorubicin induces apoptosis through mitochondria-dependent pathway.

The mitochondria membrane potential (MMP), an important parameter of mitochondrial function, was measured by flow cytometry in cells treated with decursin, doxorubicin, or both. The cotreatment significantly reduced fluorescence intensity (from 94.09% to 65.86% in U266 cells, and from 93.92% to 65.35% in MM.1S cells, implying the loss of MMP (Figure 4(d)), while either drug alone had no effect on the MMP. These results suggest that apoptosis induced by the concurrent treatment of decursin and doxorubicin is through the change of the membrane potential of the mitochondria in U266 and MM.1S cells.

### 3.4. Doxorubicin and Decursin Targeted Multiple Signaling Molecules in Multiple Myeloma Cells

**Figure 5:**
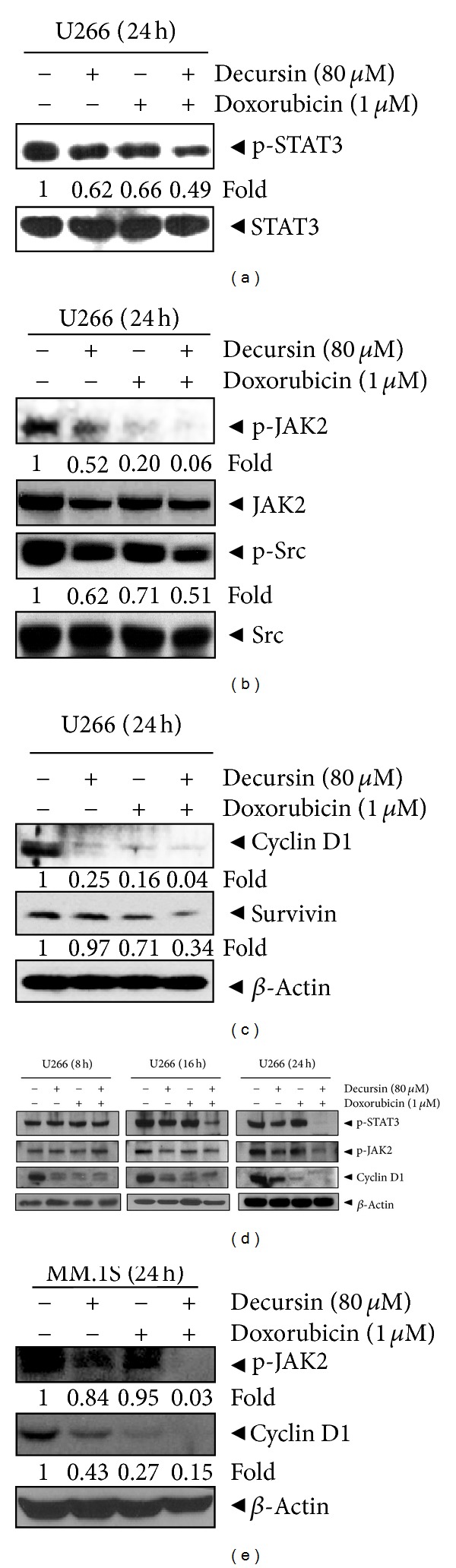
Effect of doxorubicin and decursin on STAT3 signaling in multiple myeloma cells. (a–c) U266 cells were treated with decursin (80 *μ*M) and/or doxorubicin (1 *μ*M) for 24 h. Cell lysates were prepared and subjected to western blotting for (a) phospho-STAT3 and STAT3, phospho-JAK2, JAK2, phospho-Src (b) and Src, and (c) cyclin D1 and survivin. (d) U266 cells were treated with decursin (80 *μ*M) and/or doxorubicin (1 *μ*M) for 8, 16, or 24 h. Western blotting was performed for phospho-STAT3, phospho-JAK2, and cyclin D1. (e) MM.1S cells were treated with decursin (80 *μ*M) and/or doxorubicin (1 *μ*M) for 24 h. Western blotting was performed for phospho-JAK2 and cyclin D1. A representative result of three independent experiments is shown for each experiment.

Next, we investigated whether STAT3 signaling is involved in the synergistic regulation of multiple myeloma cell survival by the cotreatment of decursin and doxorubicin. As expected, the combined treatment significantly inhibited the level of phospho-STAT3, compared to decursin or doxorubicin alone (Figure 5(a)). STAT3 is activated by the upstream kinases JAK or Src family [29] and can regulate oncogenesis by targeting various gene products [30]. Using immunoblot analysis, we showed that the combination treatment of decursin and doxorubicin dramatically suppressed the level of phospho-JAK2 and phospho-Src, compared to either drug alone (Figure 5(b)). The combination treatment also increased the magnitudes of decursin- or doxorubicin-mediated downregulation of cyclin D1 and survivin that are the products of STAT3 target genes (Figure 5(c)). The effects of decursin and doxorubicin on STAT3-related signaling molecules were also shown in cells treated for 8, 16, or 24 h (Figure 5(d)). As shown in Figure 5(d), the combination treatment suppressed pJAK2, pSTAT3, and Cyclin D1 and activated SHP-2 in a time-dependent manner in U266 cells, indicating that the synergistic effect on JAK2-STAT3-Cyclin D1 signal axis in STAT3-positive U266 cells. Conversely, the broadly acting tyrosine phosphatases inhibitor pervanadate reversed STAT3 inactivation and also PARP cleavage and caspase-3 activation induced by combined treatment of doxorubicin and decursin in U266 cells, indicating the important role of STAT3 in apoptosis induced by the combination of doxorubicin and decursin in STAT3 active U266 cells.

**Figure 6:**
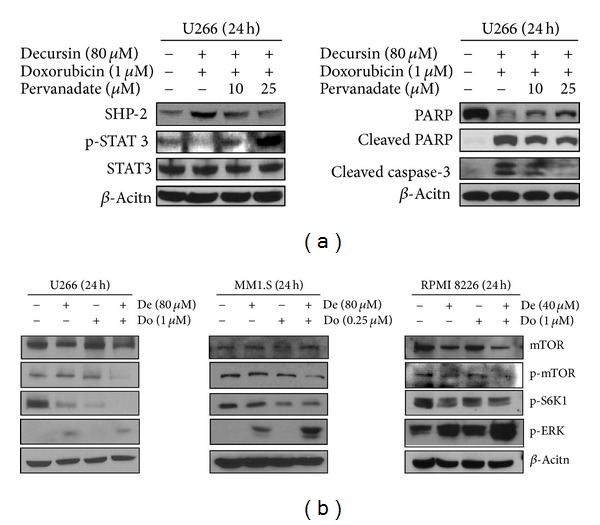
Effect of doxorubicin and decursin on mTOR pathway in multiple myeloma cells. (a) Effect of pervanadate on the STAT3 inactivation, SHP-2 activation, and cleavages of caspase-3 and PARP induced by combination of decursin and doxorubicin in U266 cells. (b) Effect of combination of decursin and doxorubicin on the phosphorylation of mTOR, S6 K1, and ERK in U266, MM1.S, and RPMI8226 cells. The cells were treated with decursin and/or doxorubicin for 24 h. Western blotting was performed with antibodies of mTOR, phospho-mTOR, phospho-S6 K1, phospho-ERK, and *β*-actin.

To explore whether the combination effect of decursin and doxorubicin is directly regulated by STAT3 signaling, parallel experiments were performed in MM.1S (STAT3 inactive) cells. As shown in Figure 5(e), the cotreatment of decursin and doxorubicin also suppressed the levels of phospho-JAK2 and cyclin D1 in MM.1S cells. However, phosphorylation of STAT3 was not observed in MM.1S cells (data not shown). In addition, to elucidate whether decursin suppression of STAT3 is associated with decursin and doxorubicin-induced apoptosis, U266 cells cotreated with decursin and doxorubicin were treated with pervanadate and expressions of SHP2, p-STAT3, PARP, and caspase-3 were then analyzed. Pervanadate treatment resulted in an increase of p-STAT3 and clearly blocked PARP cleavage, caspase-3 activation, and SHP-2 in combination of decursin and doxorubicin suggesting that decursin and doxorubicin-induced apoptosis via STAT3 inactivation in U266 cells (Figure 6(a)).

Since the combination of decursin- and doxorubicin-induced apoptosis in three multiple myeloma cells regardless of STAT3 existence, we examined another signaling pathway relevant to synergistic antitumor effect of combination of decursin and doxorubicin in three multiple myeloma cells. As shown in Figure 6(b), the combination of decursin and doxorubicin downregulated the phosphorylation of p-mTOR and its downstream S6 K1 in the three multiple myeloma cells but did not affect PI3 K and Akt signaling (data not shown). Also, the combination of decursin and doxorubicin upregulated the phosphorylation of extracellular signal-regulated kinase (ERK) but did not affect p38 and JNK (data not shown) in the three multiple myeloma cells. 

## 4. Discussion

Drug resistance is a severe problem in treating multiple myeloma, a hematologic malignant disorder [31]. Overexpression of p-glycoprotein (p-gp) [32] and the multi-drug-related protein (MRP) [33] are ones of many possible mechanisms of drug resistance in cancer therapy. The molecules allow exporting or excluding anticancer drugs, resulting in drug resistance. Various chemosensitizers or resistance modifiers such as anthracyclin (doxorubicin), vinca alkaloids (vincristine and vinblastine), and epipodophyllotoxins are chemotherapeutics that are affected by the drug efflux pump p-glycoprotein. Cell-cell and cell-stroma interactions using various adhesion molecules including very late antigen-4 (VLA-4), vascular adhesion molecule (VCAM), leukocyte function-associated antigen 1 (LFA-1), and intercellular adhesion molecule-1 (ICAM-1) [34] are important throughout myeloma pathogenesis and also contribute to drug resistance although multiple myeloma patients are initially responsive to these drugs.

The influence on the susceptibility to apoptosis is another mechanism of drug resistance to chemo or radiotherapy [35]. Several different combination regimens have been applied for the treatment of multiple myeloma. Especially, VAD regimen (vincristine (V) + doxorubicin (A) + dexamethasone (D)) is known as an efficient treatment that induces a more rapid response than other regimens for multiple myeloma [36, 37]. However, serious side effects such as myelotoxicity, neurotoxicity, and nausea still remain problematic for multiple myeloma treatment [38]. Thus, modified or novel combination regimen(s) will be necessary to improve the tolerability and efficacy of multiple myeloma therapy. For this purpose, natural compounds are ideal materials for developing new combination regimens for multiple myeloma, since most of these compounds are less or non toxic to normal cells, but able to target cancer cells specifically. For example, curcumin in combination with bortezomib synergistically induced apoptosis in human multiple myeloma U266 cells [39]. Capsaicin also significantly stimulated the apoptotic effects of Velcade and thalidomide in multiple myeloma cells [40].

In this regards, we investigated that decursin synergistically augmented apoptosis induction in its combination with doxorubicin, a component of VAD regimen, in U266 multiple myeloma cells. Several previous papers reported decursin-induced apoptosis in cancer cells. Decursin inhibited growth of human bladder and colon cancer cells via the induction of apoptosis, of G1-phase arrest, and activation of extracellular signal-regulated kinase [13]. Decursin suppressed human androgen-independent PC3 prostate cancer cell proliferation by promoting the degradation of beta-catenin [14]. We recently found that decursin-mediated apoptosis via inhibition of cyclooxygenase-2- (COX-2-) dependent survivin expression in human myeloid leukemia cells [12].

In the present study, we found that the combined treatment of decursin and doxorubicin synergistically elevated levels of the magnitude of apoptosis in human multiple myeloma cells, while it shows weak cytotoxicity in normal peripheral blood leukocytes (PBLs). The results obtained from cell viability, TUNEL, and live/dead assays were in a good agreement, suggesting that the cotreatment is able to increase the anticancer activity against multiple myeloma cells. In this apoptotic process, caspase family, aspartate-specific cysteine proteases, played a central role [41]. Activation of caspase-3 and cleavage of its substrates such as PARP and lamin A are the hallmarks of apoptosis [42]. In our results, the combination treatment augmented the levels of the active forms of caspase-3 and -9, as well as of PARP cleavage. In addition, the combination treatment drastically induced the loss of MMP. Therefore, the results further confirmed the synergy of decursin and doxorubicin in the induction of apoptosis in multiple myleoma cells.

STAT is a family of six different transcription factors that play crucial roles in cytokine signaling [43]. STAT3 is often constitutively activated in various types of human cancer including multiple myeloma and closely associated with cancer cell proliferation and antiapoptosis [44–46]. It has been well known that the growth of multiple myeloma cells is regulated by signal transduction through the JAK/STAT pathway [24]. Thus, STAT3 has been implicated as a potential therapeutic target for multiple human cancer [47]. Kim and colleagues recently reported that decursin antagonized STAT3 signaling for the sensitization of U266 cells to apoptosis [17]. In the present study, combination of decursin and doxorubicin suppressed the phosphorylation of JAK2 and Src, which are upstream protein tyrosine kinases of the STAT pathway, and STAT3 in STAT3-positive U266 cells. There are evidences that STAT3 activation is negatively regulated by protein tyrosine phosphatases (PTPs) including SHP-1, SHP-2, PTEN, PTP-*ε*, and SOCS-1 [48, 49]. Here, the combination of decursin and doxorubicin activated SHP-2 in U266 cells. Conversely, pervanadate (a general PTP inhibitor) significantly reversed STAT3 inactivation induced by combination of decursin and doxorubicin in U266 cells, supporting an important role of the PTP in dephosphorylation of STAT3 in U266 cells. Also, we found that the cotreatment of decursin and doxorubicin synergistically reduced mitochondrial membrane potential and attenuated the expression of cyclind-D1 in U266 cells, while the combination treatment downregulated the phosphorylation of JAK2 and attenuated the expression of cyclinD1 in STAT3 inactive MM1S cells, implying STAT3 independent pathway.

Given that the combination of decursin and doxorubicin induced cytotoxicity or apoptosis in three multiple myeloma cells regardless of STAT3 existence, another signaling pathway can be involved in the synergistic antitumor effect of combination of decursin and doxorubicin in three multiple myeloma cells. The mTOR regulates the various cellular processes such as cell growth, angiogenesis, and survival [50] through the phosphorylation of P70S6 K [51]. Also, mTOR regulates the cell proliferation through controlling the production of cyclin D1 [52] Thus, several clinical studies revealed that inhibition of mTOR might be a good target for cancer therapy [20, 53] and several drugs such as everolimus, ridaforolimus, and temsirolimus as mTOR inhibitors have been used for clinical trials [54–56]. In the current study, the phosphorylation of mTOR and S6K1 was inhibited by combination of decursin and doxorubicin in three multiple myeloma cells, implying that combination of decursin and doxorubicin induces apoptosis via mTOR/S6K1 pathway. Also, with the evidence that loss of mTOR resulted in an activation of ERK [57], the combination of decursin and doxorubicin upregulated the phosphorylation of ERK in the three multiple myeloma cells, indicating that ERK activation is associated with inhibition of mTOR/S6K1 signaling.

Anticancer activity of doxorubicin has been explained by inhibiting DNA polymerase [58] and topoisomerase II [59]. Spatial or temporal distribution of the enzymes has been reported during cancer cell proliferation [60, 61]. Indeed, our FACS analysis data using PI staining showed anti-proliferation activity of decursin and doxorubicin. Similarly, several studies reported that anti-cancer activity of doxorubicin was significantly increased in combination of other drugs such as all-*trans *retinoid (ATR) [62] and artesunate [63]. It can be assumed that synergistic anticancer effect of decursin and doxorubicin may result partially from inhibiting DNA polymerase or topoisomerase II activities in addition to the suppression of STAT3 activation. Thus, additional experiments are required to verify this hypothesis in the near future. 

## 5. Conclusions

In summary, our results demonstrate the synergistic effect of decursin and doxorubicin on the induction of apoptosis via the inhibition of mTOR and/or STAT3 signaling pathway in multiple myeloma cells. Notably, the synergy of decursin and doxorubicin was verified by calculating CI value (CI < 1). Thus, our findings propose that the combination of decursin and doxorubicin may be beneficial for the improvement of the treatment of multiple myeloma patients.
